# Distinct PD-L1/PD1 Profiles and Clinical Implications in Intrahepatic Cholangiocarcinoma Patients with Different Risk Factors

**DOI:** 10.7150/thno.36276

**Published:** 2019-07-09

**Authors:** Jia-Cheng Lu, Hai-Ying Zeng, Qi-Man Sun, Qing-Nan Meng, Xiao-Yong Huang, Peng-Fei Zhang, Xuan Yang, Rui Peng, Chao Gao, Chuan-Yuan Wei, Ying-Hao Shen, Jia-Bing Cai, Rui-Zhao Dong, Ying-Hong Shi, Hui-Chuan Sun, Yujiang G. Shi, Jian Zhou, Jia Fan, Ai-Wu Ke, Liu-Xiao Yang, Guo-Ming Shi

**Affiliations:** 1 Department of Liver Surgery and Transplantation, Liver Cancer Institute, Zhongshan Hospital, Fudan University; Key Laboratory of Carcinogenesis and Cancer Invasion (Fudan University), Ministry of Education, Shanghai 200032, P.R. China; 2 Cancer Center, Institutes of Biomedical Sciences, Fudan University, Shanghai 200031, P.R. China; 3 Department of Pathology, Zhongshan Hospital, Fudan University, Shanghai 200032, P.R. China; 4 Division of Endocrinology, Diabetes and Hypertension, Departments of Medicine, Brigham and Women's Hospital, Harvard Medical School, Boston, MA 02115, USA; 5 Department of Critical Care Medicine, Zhongshan Hospital, Fudan University, Shanghai 200032, P.R. China

**Keywords:** intrahepatic cholangiocarcinoma, hepatitis B virus, hepatolithiasis, programmed cell death protein 1, programmed cell death protein ligand 1, immune checkpoint blockage

## Abstract

**Rationale**: PD1/PD-L1 immune checkpoint inhibitors have shown promising results for several malignancies. However, PD1/PD-L1 signaling and its therapeutic significance remains largely unknown in intrahepatic cholangiocarcinoma (ICC) cases with complex etiology.

**Methods**: We investigated the expression and clinical significance of CD3 and PD1/PD-L1 in 320 ICC patients with different risk factors. In addition, we retrospectively analyzed 7 advanced ICC patients who were treated with PD1 inhibitor.

**Results**: The cohort comprised 233 patients with HBV infection, 18 patients with hepatolithiasis, and 76 patients with undetermined risk factors. PD-L1 was mainly expressed in tumor cells, while CD3 and PD1 were expressed in infiltrating lymphocytes of tumor tissues. PD1/PD-L1 signals were activated in tumor tissues, and expression was positively correlated with HBV infection and lymph node invasion. More PD1^+^ T cells and higher PD-L1 expression were observed in tumor tissues of ICC patients with HBV infection compared to patients with hepatolithiasis or undetermined risk factors. More PD1^+^ T cells and/or high PD-L1 expression negatively impacted the prognosis of patients with HBV infection but not those with hepatolithiasis. Multivariate analysis showed PD1/PD-L1 expression was an independent indicator of ICC patient prognosis. Advanced ICC patients with HBV infection and less PD1^+^ T cells tended to have good response to anti-PD1 therapy.

**Conclusion**: Hyperactivated PD1/PD-L1 signals in tumor tissues are a negative prognostic marker for ICCs after resection. HBV infection- and hepatolithiasis-related ICCs have distinct PD1/PD-L1 profiles. Further, PD1^+^ T cells could be used as a biomarker to predict prognosis and assay the efficiency of anti-PD1 immunotherapy in ICC patients with HBV infection.

## Introduction

Intrahepatic cholangiocarcinoma (ICC) is the second most common primary liver cancer after hepatocellular carcinoma (HCC) 1, 2. ICC incidence is increasing worldwide because of highly prevalent risk factors, such as hepatitis B virus (HBV), hepatitis C virus, hepatolithiasis, and hepatobiliary flukes 3, 4. Few treatments can significantly improve the clinical prognosis of patients with advanced and unresectable ICC 5. Due to early local invasion, distant metastasis, and lack of effective treatment, ICC prognosis remains dismal 5-7. Usually, curative resection is an option for only a minority of ICC patients. However, even radical resection can seldom help patients acquire a median survival of >30 months. Therefore, new and efficient treatments for ICC patients are needed 6.

Recently, immune checkpoint molecule programmed cell death protein 1 (PD1)/programmed cell death protein ligand 1 (PD-L1)-based inhibitors have shown promising results in several malignant tumors 8-12. As an important immune checkpoint, PD1 is expressed on multiple immune cells and plays a negative regulatory role in antigen response 13, 14. PD-L1 (also called B7-H1), a binding and functional partner of PD1, suppresses T cell responses after interacting with PD1, especially in the tumor microenvironment 15, 16. Aberrant PD-L1 expression is detected in many malignancies, such as non-small cell lung cancer, renal cell carcinoma, Hodgkins lymphoma, and HCC 14, 17, 18. Moreover, hyper-activated PD1/PD-L1 expression is considered a marker to predict prognosis and assay the tumor response of anti-PD1 immunotherapy in several malignancies 19, 20.

A recent study reported that PD-L1 is overexpressed in occupational cholangiocarcinoma, a type of cholangiocarcinoma arising from occupational exposure to organic solvents, indicating that PD1/PD-L1 axis-dependent immune escape may be an important mechanism for formation of occupational cholangiocarcinoma 21. A small sample study of 31 ICCs reported that PD1/PD-L1 expression is upregulated in tumor tissues and is correlated with tumor differentiation and pTNM stage 22. Another study of 58 ICCs showed the PD-L1 is mainly expressed in inflammatory cells 23. Further, a study of 54 ICC patients showed that high PD-L1 expression in tumors is associated with lower overall survival 24. However, because ICCs are characterized by tumor heterogeneity and complex etiology 25, 26, these small sample studies provide an incomplete analysis of the role of PD1/PD-L1 in ICC. Therefore, we analyzed expression profiles of the PD1/PD-L1 axis in tumor tissues from a large cohort of ICC patients.

## Methods

### Study population

We enrolled 320 consecutive ICC patients who underwent curative resection from 2005-2011 at the Liver Cancer Institute, Zhongshan Hospital, Fudan University. All enrolled patients met the following inclusion and exclusion criteria: (1) patients were pathologically diagnosed with ICC; and (2) patients were not administered any anti-cancer treatments before surgery. Histopathological diagnosis of ICC was based on World Health Organization criteria 27. Follow-up data were collected until October 31, 2016.

Data from another 7 advanced and unresectable ICC patients who were administered anti-PD1 immunotherapy (Nivolumab, 3 mg/kg, once every two weeks) >4 times from 2017-2018 were collected and recorded, and tumor response was assayed according to RECIST1.1 28. Written informed consent was obtained from each patient. This study was approved by the Zhongshan Hospital Research Ethics Committee (Y2017-130).

### Tissue microarray and immunohistochemistry

A tissue microarray (TMA) was constructed by Shanghai Biochip Co. Ltd. (Shanghai, China), as described previously 29. Immunohistochemistry (IHC) was performed with Leica BOND-MAX system (Wetzlar, Germany), as described previously 30. Primary antibodies used were: monoclonal rabbit anti-human PD-L1 (dilution 1:100; #SP142, GeneTech Co. Ltd., Shanghai, China), monoclonal rabbit anti-human CD3 (dilution 1:500; #NCL-L-CD3-565, Leica, Cambridge, UK), and monoclonal rabbit anti-human PD1 antibody (dilution 1:500; #ab137132, Abcam, Cambridge, UK). Images were captured with Leica Q Win Plus v3 software (Leica Microsystems Imaging Solutions, Cambridge, UK). ICC diagnosis was validated by H&E staining.

### Quantification of CD3, PD1, and PD-L1 expression

Infiltrating T cells positively stained for CD3 and PD1 in each 1-mm-diameter TMA core were counted manually by two independent pathologists under high-power magnification (200×). For the 7 ICC patients treated with PD1 inhibitor, positive staining for CD3 and PD1 T cells were counted in 5 high-power fields (200×), and average number of positive-stained T cells was converted into an equivalent cell number for a TMA core (0.95×n/0.785) and recorded as final number of positive-stained T cells. To assay PD-L1 positive staining, we used a scoring method to calculate the rank of PD-L1 expression, as described previously with minor revisions 31. Tumor cell proportion score was on a 6-point scale to indicate the percentage of stained tumor cells: a score of 1 indicated <1% of the tissue section; a score of 2 indicated 1%-4% of the tissue section; a score of 3 indicated 5%-9% of the tissue section; a score of 4 indicated 10%-24% of the tissue section; a score of 5 indicated 25%-49% of the tissue section; and a score of 6 indicated ≥50% of the tissue section (**Supplementary Figure 1**). The median was selected as the cutoff value for high or low PD1 expression. By calculating Youden Index of PD-L1 staining, >5% positive area of PD-L1 expression in tumor cells was defined as positive, as described previously 24.

### Statistical analysis

Statistical analysis was performed using SPSS 20.0 (Chicago, IL). Values were recorded as mean ± standard deviation. T-test, Paired t-test, Wilcoxon signed-rank test, χ^2^-test, Fisher's exact test, and Wilcoxon rank-sum test were adopted to analyze correlation between target markers and clinicopathologic variables. Survival curves were estimated by the Kaplan-Meier method, and significance was verified by log-rank test. Cox proportional hazards models were developed to determine the association of clinical characters with survival. Relative risks were expressed as hazard ratios (HRs) with 95% confidence intervals (CIs). *p* < 0.05 (two-tailed) was considered statistically significant.

## Results

### Patient clinicopathologic profiles

A total of 320 patients were enrolled in the study, including 191 (59.7%) males and 129 (40.3%) females. The median age was 58 years. In the cohort, 233 (72.8%) patients had HBV infection, 85 (26.6%) patients had cirrhosis, and 18 (5.6%) patients had hepatolithiasis. No patients had abnormal body mass index (BMI) or history of hepatitis C virus infection. Among 160 patients with recorded HBV DNA copy number, 16.9% (27/160) were positive for HBV DNA (above minimum detection level). In total, 155 (48.4%) patients had elevated serum CA19-9 (≥37 ng/mL); 40 (12.5%) patients had elevated preoperative α-fetoprotein (≥20 ng/mL), and 23 (7.2%) patients had elevated preoperative alanine transaminase (ALT) (≥75 U/L). Further, 309 (96.6%) patients were classified as Child-Pugh grade A, and 3.4% of patients were Child-Pugh grade B.

Regarding tumors, 76.6% (245/320) of patients had a solitary tumor, 55.0% (176/320) of patients had large tumors (>5 cm), and 88.4% (283/320) of tumors were non-encapsulated. In total, 77.5% (248/320) of patient tumors were TNM stage I or II, while 22.5% of patient tumors were TNM III. Further, 60.9% of tumors had stage I or II differentiation, and 39.1% of tumors had stage III or IV differentiation. In addition, 16.9% (54/320) of patients had lymph node invasion, and 15.0% (48/320) of patients had microvascular invasion. Clinicopathologic profiles are summarized in **Supplementary Table 1**.

### Highly expressed immune checkpoint PD1/PD-L1 in ICCs

**Figure 1:**
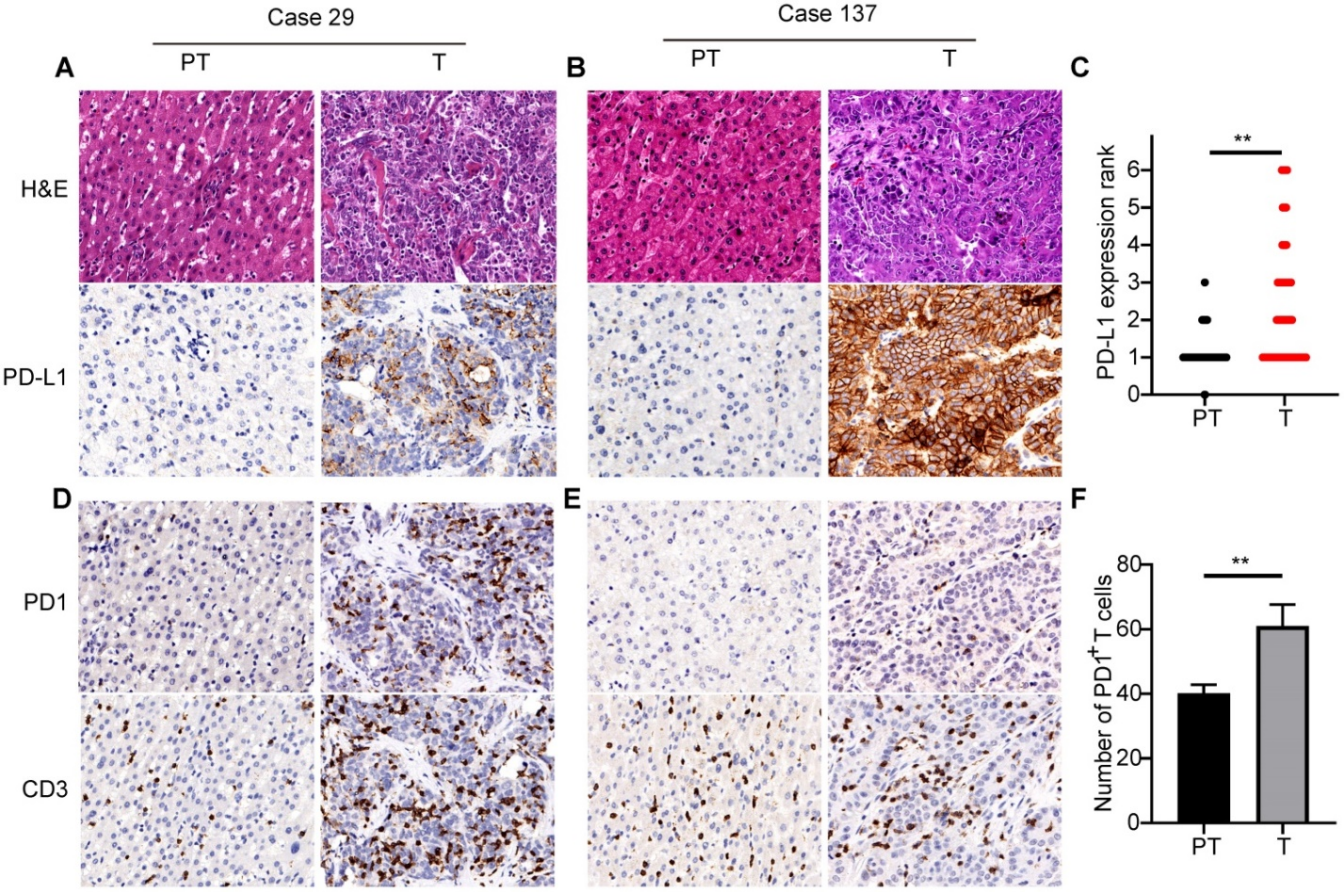
** PD1/PD-L1 expression in tumor tissues of 320 ICCs.** (**A**) and (**B**) Two representative ICC tissues showing different levels of PD-L1 expression (200×). (**C**) Semi-quantitative analysis of PD-L1 staining in tumor tissues (T) and peritumor tissues (PT) (**p < 0.01, Wilcoxon signed-rank test). (**D**) and (**E**) Two representative ICC tissues with different numbers of PD1- and CD3-positive cells (200×). (**F**) Statistical analysis of PD1^+^ T cells in tumor tissues (T) and peritumor tissues (PT) (**p < 0.01, paired Student's t-test).

PD-L1 expression was mostly located in the cellular membrane and cytoplasm of tumor cells (**Figure 1A**). Moreover, PD-L1 expression in tumors from different patients significantly differed in positive area and intensity (**Figure 1A and B**). Comparison of PD-L1 expression between tumor and adjacent nontumor tissues showed significantly higher expression in tumor tissues than in peritumor tissues (**Figure 1C**). Positive expression of CD3 and PD1 was detected in the cellular membrane and cytoplasm of infiltrating lymphocytes in tumor tissues and adjacent liver tissues (**Figure 1D and E**). Quantification indicated that the number of PD1^+^ T cells and CD3^+^ T cells in tumor tissues were 60.1±6.5 and 411.7±60.0, respectively. Further, there were significantly more PD1^+^ T cells in tumor tissues than in adjacent peritumor liver tissues (**Figure 1F**).

### Relationship of PD1/PD-L1 with clinicopathologic features of ICCs

PD-L1 expression was diverse in each sample (**Supplementary Figure 1**). We calculated the Youden Index of PD-L1 expression and divided the cohort into high expression (PD-L1^high^) and low expression (PD-L1^low^) subgroups according to a cut-off value of 5% positive staining. We also classified the cohort into two subgroups according to median number of PD1^+^ T cells (n = 15.5) in tumor tissues. High PD-L1 expression was positively correlated with positive HBV infection (*p* = 0.007), high TNM stage (*p* = 0.025), and lymph node invasion (*p* = 0.007) (**Table 1**). Similarly, high number of PD1^+^ T cells in tumor tissues positively correlated with positive HBV infection (*p* = 0.017), high TNM stage (*p* = 0.016), and lymph node invasion (*p* = 0.003) (**Table 1**).

### Prognostic implication of PD1/PD-L1 axis in ICC patients

Up to the last follow-up, 200 patients had tumor recurrence and 232 patients died, including 48 patients who died without tumor recurrence. The 1-, 2- and 5-year overall survival (OS) rates in the whole cohort were 73.6%, 54.6%, and 33.8%, respectively; 1-, 2- and 5-year cumulative recurrence rates were 44.9%, 55.5%, and 66.3%, respectively.

**Figure 2:**
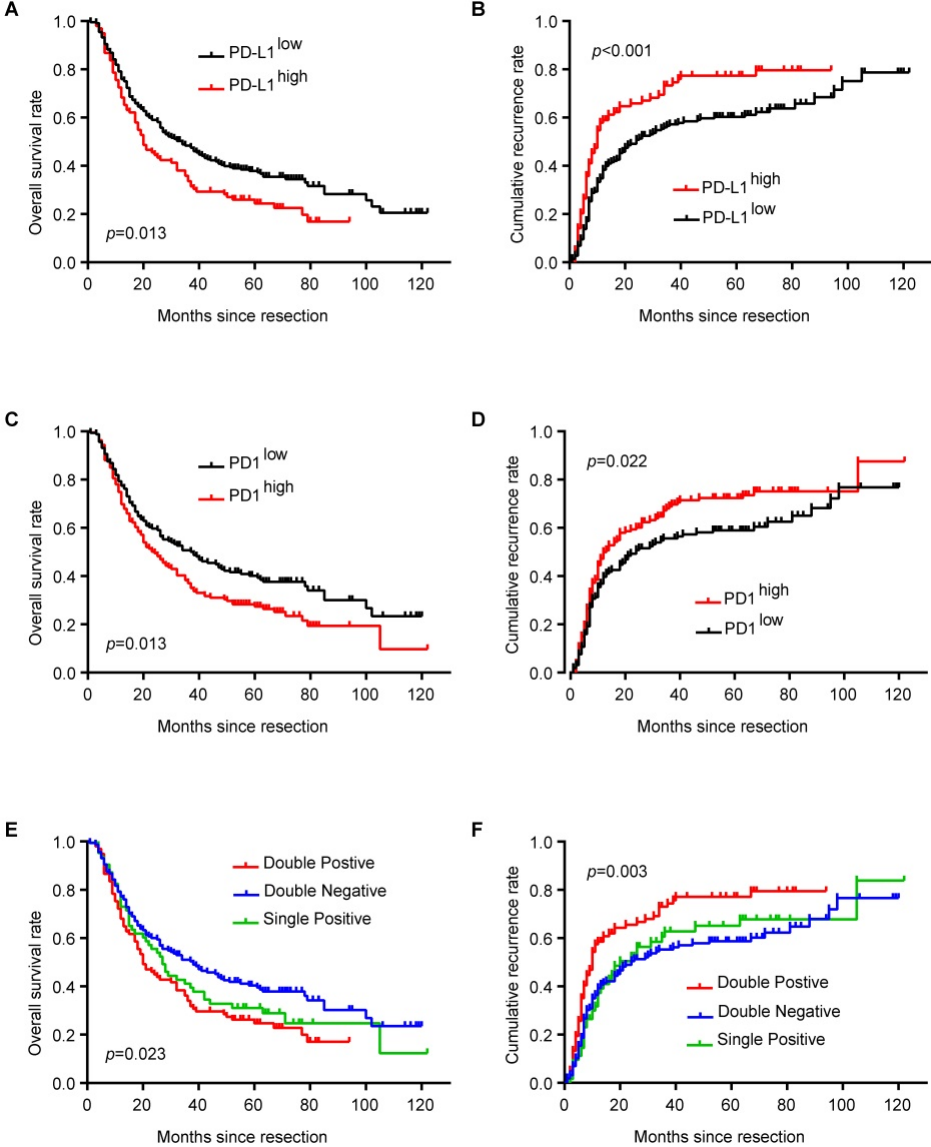
** Prognostic implication of PD1/PD-L1 axis in ICC patients.** (**A**) Kaplan-Meier estimate of overall survival in the whole cohort with different PD-L1 levels (log-rank test). (**B**) Kaplan-Meier estimate of cumulative recurrence in the whole cohort with different PD-L1 levels (log-rank test). (**C**) Kaplan-Meier estimate of overall survival in the whole cohort with different PD1 levels (log-rank test). (**D**) Kaplan-Meier estimate of cumulative recurrence in the whole cohort with different PD1 levels (log-rank test). (**E**) Kaplan-Meier estimate of overall survival in the whole cohort with combined PD1/PD-L1 levels (double-positive refers to PD1^high^/PD-L1^high^; single positive refers to PD1^high^/PD-L1^low^ or PD1^low^/PD-L1^high^; double negative refers to PD1^low^/PD-L1^low^) (log-rank test). (**F**) Kaplan-Meier estimate of cumulative recurrence in the whole cohort with combined PD1/PD-L1 levels (double positive refers to PD1^high^/PD-L1^high^; single positive refers to PD1^high^/PD-L1^low^ or PD1^low^/PD-L1^high^; double negative refers to PD1^low^/PD-L1^low^) (log-rank test).

PD-L1^high^ ICC patients had shorter OS (*p* = 0.013) and higher cumulative recurrence rate (*p* < 0.001) than PD-L1^low^ patients (**Figure 2A and B**). Similarly, ICC patients with more PD1^+^ T cells in tumor tissues had shorter OS (*p* = 0.013) and higher cumulative recurrence rate (*p* = 0.022) than patients with less PD1^+^ T cells (**Figure 2C and D**). Considering the combined role of PD1 and PD-L1 in regulating T cell function, ICC patients with high expression of both PD1 and PD-L1 had the poorest prognosis among patients with high expression of only PD1 or PD-L1 and patients with low expression of both PD1 and PD-L1 (**Figure 2E and F;** OS, *p* = 0.023; cumulative recurrence rate, *p* = 0.003).

Univariate analysis showed that large tumor size (>5 cm), multiple tumors, high TNM stage, and lymph node invasion/microvascular invasion were risk factors for OS and cumulative recurrence rate, while hepatolithiasis was a risk factor for OS (**Table 2**). Interestingly, PD1 and PD-L1 expression in tumor tissues also correlated with OS and cumulative recurrence rate.

These risk factors from univariate analysis were adopted as covariates in a multivariate Cox proportional hazards model. PD-L1, multiple tumors, and lymph node invasion/microvascular invasion were independent prognostic indicators for cumulative recurrence rate. With combined expression of PD1/PD-L1, the hyperactivated PD1/PD-L1 axis was also an independent prognostic predictor for both OS (*p* = 0.031) and cumulative recurrence rate (*p* = 0.001) (**Table 2**).

### Distinct profiles and implication of PD1/PD-L1 expression in ICC patients with different risk factors

Epidemiologic data show that HBV infection and hepatolithiasis are two important risk factors for ICC incidence 3, 32. In the entire cohort, 7 patients (2.1%) had both HBV infection and hepatolithiasis, 226 patients (70.6%) had only HBV infection, 11 patients (3.4%) had only hepatolithiasis, and 76 patients had undetermined risk factors (without HBV infection or hepatolithiasis).

Similar to expression of PD1^+^ T cells in the whole cohort, ICC patients with only HBV infection had more PD1^+^ T cells in tumor tissues than in corresponding liver tissues (68.0±8.9 vs 45.5±3.4, respectively; *p* = 0.001). Further, tumor tissues from ICC patients with HBV infection had 68.0±8.9 PD1^+^ T cells, significantly more than those from patients with hepatolithiasis (9.2±2.5; *p* < 0.001) and patients with undetermined risk factors (42.4±7.4; *p* = 0.027). Patients with HBV infection also had higher expression of PD-L1 in tumor tissues than in corresponding liver tissues, similar to the whole cohort (*p* < 0.001). ICC patients with only HBV infection had higher frequency of high PD-L1 expression (34.5%) compared to patients with hepatolithiasis (0%, *p* = 0.018) or patients with undefined risk factors (22.4%, *p* = 0.049).

Unexpectedly, all 11 patients with only hepatolithiasis had less PD1^+^ T cells and lower PD-L1 expression in tumor tissues, distinct from patients with only HBV infection. Although there was tendency for higher PD-L1 expression in the tumor tissues of ICC patients with hepatolithiasis compared to corresponding liver tissues, no significant difference was observed (*p* = 0.317). However, patients with hepatolithiasis had less PD1^+^ T cells in tumor tissues than in corresponding liver tissues (9.2±2.5 vs 33.5±8.0, respectively; *p* = 0.018).

**Figure 3:**
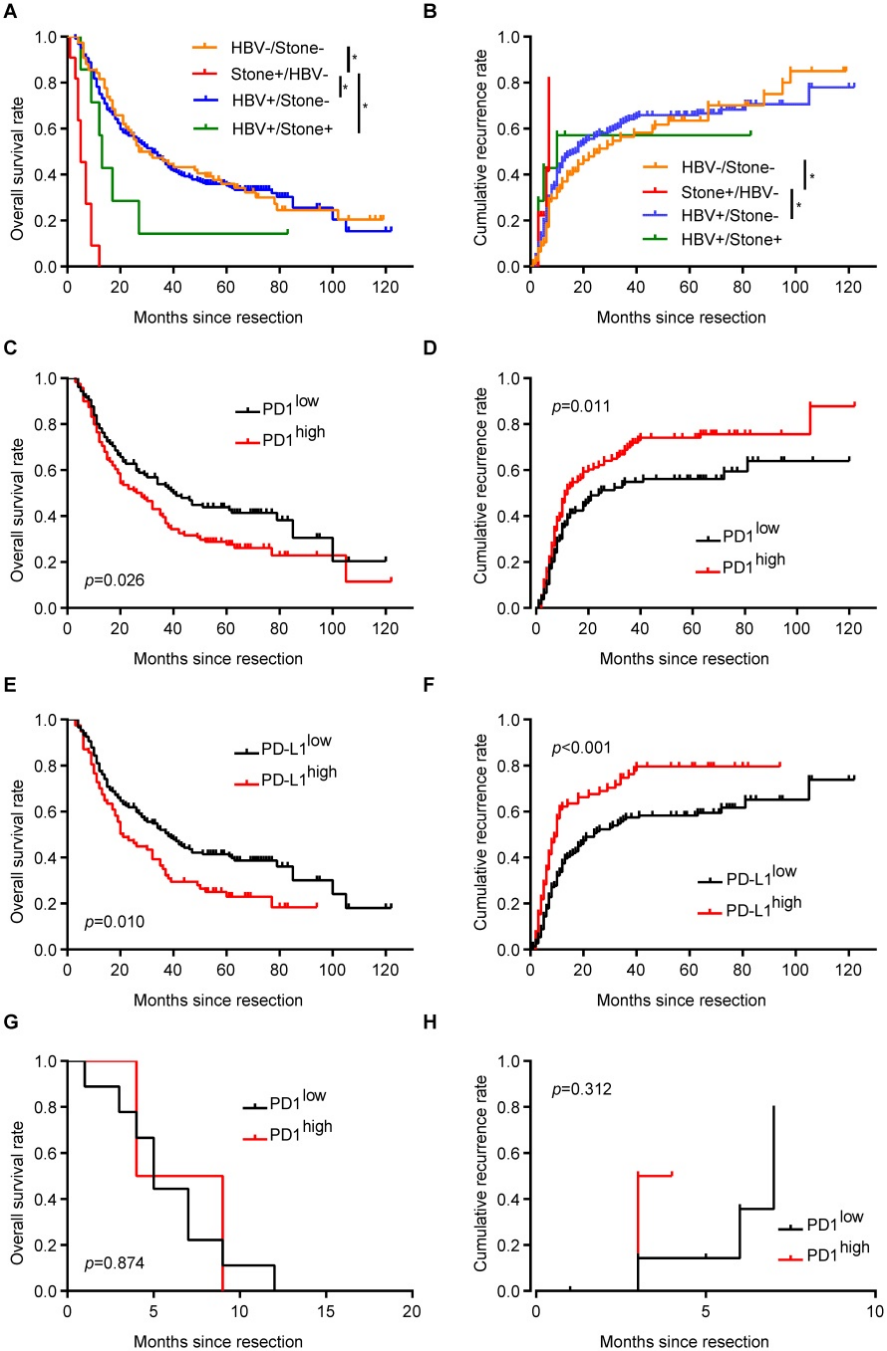
** Implication of PD1/PD-L1 expression in ICC patients with different risk factors.** (**A**) Kaplan-Meier estimate of overall survival in the whole cohort with different risk factors (*p < 0.05, log-rank test). (**B**) Kaplan-Meier estimate of cumulative recurrence in the whole cohort with different risk factors (*p < 0.05, log-rank test). (**C**) Kaplan-Meier estimate of overall survival of ICC patients with only HBV infection, grouped by PD1 level (log-rank test). (**D**) Kaplan-Meier estimate of cumulative recurrence in ICC patients with only HBV infection, grouped by PD1 level (log-rank test). (**E**) Kaplan-Meier estimate of overall survival of ICC patients with only HBV infection, grouped by PD-L1 level (log-rank test). (**F**) Kaplan-Meier estimate of cumulative recurrence in ICC patients with only HBV infection, grouped by PD-L1 level (log-rank test). (**G**) Kaplan-Meier estimate of overall survival of ICC patients with only hepatolithiasis, grouped by PD1 level (log-rank test). (**H**) Kaplan-Meier estimate of cumulative recurrence in ICC patients with only hepatolithiasis, grouped by PD1 level (log-rank test).

ICC patients with hepatolithiasis had the poorest prognosis in term of OS and cumulative recurrence rate, while there were no survival differences between patients with HBV infection and patients with undetermined risk factors (**Figure 3A and B**). For ICC patients with only HBV infection, patients with more PD1^+^ T cells in tumor tissues had shorter OS (*p* = 0.026, **Figure 3C**) and higher cumulative recurrence rates (*p* = 0.011, **Figure 3D**) than patients with less PD1^+^ T cells. Similarly, PD-L1^high^ patients had a worse prognosis than PD-L1^low^ patients (OS, *p* = 0.010, **Figure 3E;** cumulative recurrence rate, *p* < 0.001, **Figure 3F**).

For ICC patients with only hepatolithiasis, PD1 expression showed no significance in term of OS (**Figure 3G**, *p* = 0.874) and cumulative recurrence rate (**Figure 3H**, *p* = 0.312). For ICC patients with undetermined risk factors, neither PD1 nor PD-L1 expression had an effect on patient prognosis (**Supplementary Figure 2A-D**).

### Positive response to anti-PD1 immunotherapy in ICC patients with HBV infection and low PD1^+^ T cells

**Figure 4:**
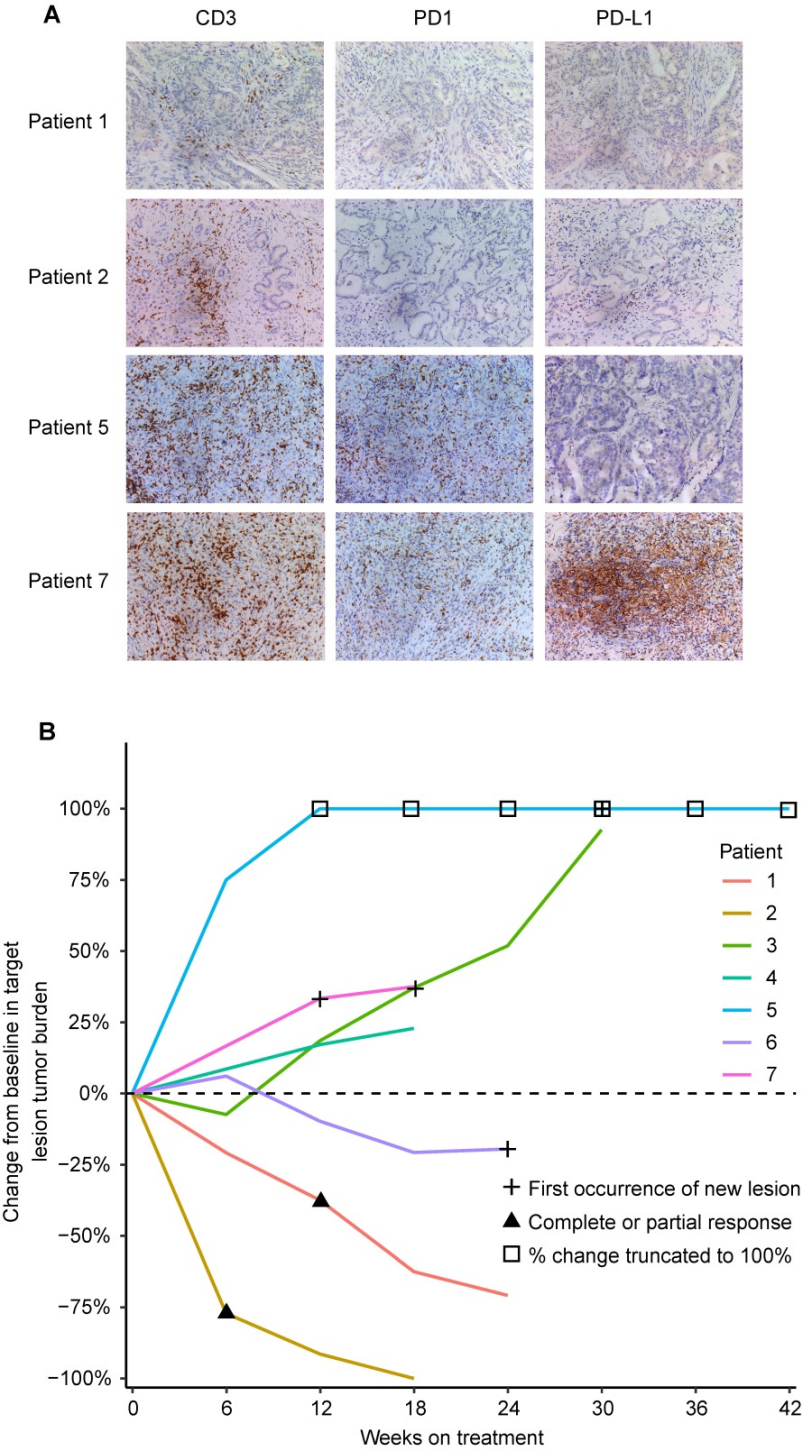
** CD3, PD1, and PD-L1 expression and tumor responses to anti-PD1 immunotherapy in 7 advanced ICC patients.** (**A**) Representative ICC tissues stained for CD3, PD1, and PD-L1 (200×). (**B**) Percentage change in tumor burden after anti-PD1 immunotherapy.

We retrospectively analyzed the relationship between clinicopathological characteristics including expression of the PD1/PD-L1 axis and tumor response in 7 advanced and unresectable ICC cases with HBV infection and treated with anti-PD1 immunotherapy. Clinicopathological characteristics are listed in **Supplementary Table 2**. Among 5 ICC patients with high PD1^+^ T cells (>19 cells/200×), 4 patients had evident tumor progression, and 1 patient presented stable disease after three cycles of anti-PD1 immunotherapy (**Figure 4A and B**). Unexpectedly, 2 patients with low PD1^+^ T cells (≤19 cells/200×) showed evident tumor shrinkage after three cycles of anti-PD1 immunotherapy (**Figure 4A and B**).

## Discussion

Our data show that PD1/PD-L1 signals are hyper-activated in tumor tissues of a large cohort of ICCs. Elevated PD1/PD-L1 signals were positively correlated with malignant phenotypes, such as lymph node invasion and high TNM stage. Further, hyper-activated PD1/PD-L1 signals in tumor tissues were an independent marker for patient prognosis—ICC patients expressing high levels of PD1/PD-L1 signals had the poorest prognosis among the entire cohort. Therefore, based on a large sample, we provide convincing evidence that hyper-activated PD1/PD-L1 signals play a substantial role in the development and progression of ICC.

Another important finding from this study is the distinct PD1/PD-L1 profiles in ICCs with different risk factors. Multiple epidemiological data show that HBV infection and hepatolithiasis are two main risk factors for ICCs 32-34. These risk factors result in continuous liver inflammation, imbalance of immune homeostasis, and tumorigenesis 33, 35-37. Our results revealed that PD1/PD-L1 signals were hyper-activated in tumor tissues of ICC patients with HBV infection but not in ICC patients with hepatolithiasis, who had evidently downregulated PD1/PD-L1 signals. Moreover, PD1/PD-L1 expression in tumors of ICC patients with HBV infection was stronger than in corresponding liver tissues. In addition, ICC patients with HBV infection and overexpressed PD1/PD-L1 signals had poorer prognosis than patients expressing low PD1/PD-L1 signals, although we did not find the same results in ICC patients with hepatolithiasis or patients with undetermined risk factors. These results indicate that hyper-activated PD1/PD-L1 signaling may play a key role in the development and progression of ICC with HBV infection.

Our results show that PD1^+^ T cells were rich in the tumor microenvironment of ICC patients with HBV infection. During chronic infection, these PD1^+^ T cells represent an exhausted state of T cells 38, probably leading to decreased tumor responses to anti-PD1 immunotherapy, as relatively low tumor response to PD1 inhibitor pembrolizumab is observed in HCC with HBV infection or HCV infection 28. It is well-established that both pre- and postoperative antiviral therapies effectively prolong OS of HBV-infected ICC patients 39. A recent study also indicates that a population of PD1^+^ T cells is specially present in HBV-infected patients who successfully discontinue treatment without hepatic flare 40. In the present study, only 13.7% patients with HBV infection had high preoperative HBV DNA levels (≥2,000 IU/mL). However, our study found no significant relationship between preoperative HBV DNA level and PD1/PD-L1 signals. Therefore, these PD1^+^ tumor infiltrating lymphocytes (TILs) in ICC patients with HBV infection probably have lost their T cell function and maybe act as a marker for assaying tumor response of PD1 inhibitor 11. In the context of several malignancies, such as lung cancer, the expression of PD-L1 showed a good predictor for assaying tumor response of PD1 inhibitor 41. However, the expression of PD-L1 in tumor cells of HCC could not act as a predictor for assaying tumor response of PD1 inhibitor, which was probably contributed to distinct liver microenvironment 15. In present studies, 233 (72.8%) ICC patients had HBV infection, and the number of PD1^+^ T cells in the tumor tissues from ICC patients only with HBV infection was significantly more than those from patients with hepatolithiasis and those from patients with undefined risk factor. Theoretically, PD1 inhibitor can targeted PD-L1/PD-1 immune checkpoint to reactivate an antitumor response. However, in the context of liver microenvironment characterized by high PD1^+^ TIL cells in HBV-related patients, PD1 inhibitor is probably not enough to block PD-L1/PD1 immune checkpoint. A recent study also showed that blocking PD1/PD-L1 only increased the frequency of tumor-specific T cells in HCC patients but did not restore T cell function 42. Therefore, the PD-L1 expression did not act as a marker for assaying tumor response of PD1 inhibitor for HCC patients and ICC patients with HBV infection. In addition, tumor response was also influenced by other factors, such as tumor mutation burden 43, and the expression of other immune check points (TIM-3, CTLA-4) 13.

Importantly, our retrospective observation revealed that 2 ICC patients with HBV infection and low PD1^+^ TILs had a good response to anti-PD1 immunotherapy, although these data needed to be further validated by future randomized controlled trials. Similarly, our results also demonstrate that PD-L1 is overexpressed in the tumor tissues of ICC patients with HBV infection. Usually, PD-L1 expressed in tumor cells interacts with PD1 in T cells, activates the PD1/PD-L1 signal, and negatively regulates the immune reaction 44. As such, high PD-L1 expression in ICC patients with HBV infection may induce tumor cells to evade immune surveillance, resulting in a shortened OS and high recurrence rate after curative resection.

In the present study, PD1/PD-L1 expression in tumor tissues did not correlate with the risk factor of hepatolithiasis. Moreover, PD1/PD-L1 expression did not influence the prognosis of ICC patients with hepatolithiasis. These distinct PD1/ PD-L1 profiles indicate that other immune checkpoint molecules may play a key role in immune regulation in hepatolithiasis- related ICC 45. Several studies have showed that the liver immune microenvironments between patients with hepatolithiasis and patients with HBV infection are distinct 37, 46. Neutrophil infiltration is usually observed in livers of patients with hepatolithiasis 47, which represents an immune reaction to bacteria. But for patients with HBV infection, lymphocytes rather than neutrophils act as scavengers for virus infection 48. Different inflammatory cells and cytokines in ICC patients with HBV infection or hepatolithiasis probably result in distinct immune microenvironments including different profiles of PD1/PD-L1, and patients' outcomes.

The significance of our research is that it not only provides a biomarker to predict the prognosis of ICC patients and assay the tumor response to anti-PD1 immunotherapy, but it also provides a new immunotherapeutic strategy for ICC with different risk factors. However, our study also has some limitations: (1) our results need to be validated by a randomized, controlled study; (2) immune checkpoint molecules in ICC patients with hepatolithiasis need to be identified; and (3) immune profiles of other risk factor-related ICCs need to be uncovered.

In conclusion, our study shows that hyper-activated PD1/PD-L1 signals in the tumor tissues of ICC patients with HBV infection play a substantive role in disease development and progression. Further, distinct PD1/PD-L1 profiles may be a biomarker to predict the prognosis and assay tumor response to PD1 inhibitor in ICC patients with HBV infection.

## Supplementary Material

Supplementary figures and tables.

## Figures and Tables

**Table 1:** Correlation of clinicopathologic characteristics with tumor PD-L1/PD1 expression

Characteristics	PD-L1				PD1		
	Low	High	*p* value		Low	High	*p* value
**Age(y)**							
≤58	117	52	0.945		83	86	0.737
>58	104	47			77	74	
**Gender**							
Male	126	65	0.145		88	103	0.087
Female	95	34			72	57	
**HBV infection**							
Negative	70	17	0.007		53	34	0.017
Positive	151	82			107	126	
**HBV DNA copy number**							
Negative	89	44	0.509		62	71	0.398
Positive	17	10			12	15	
Undetermined	115	45			86	74	
**Hepatolithiasis**							
Negative	207	95	0.41*		150	152	0.627
Positive	14	4			10	8	
**Liver cirrhosis**							
Negative	168	67	0.118		124	111	0.1
Positive	53	32			36	49	
**AFP (ng/mL)**							
<20	191	89	0.385		136	144	0.176
≥20	30	10			24	16	
**CA19-9 (U/mL)**							
<37	114	51	0.991		89	76	0.146
≥37	107	48			71	84	
**ALT (U/L)**							
<75	204	93	0.601		150	147	0.516
≥75	17	6			10	13	
**Child-Pugh stage**							
A	216	93	0.085*		157	152	0.125
B	5	6			3	8	
**Tumor number**							
Single	175	70	0.098		126	119	0.356
Multiple	46	29			34	41	
**Tumor size (cm)**							
≤5	96	48	0.402		69	75	0.500
>5	125	51			91	85	
**Tumor differentiation**							
I/II	140	55	0.187		102	93	0.302
III/IV	81	44			58	67	
**TNM stage**							
I/II	179	69	0.025		133	115	0.016
III	42	30			27	45	
**Microvascular invasion**							
Negative	187	85	0.773		140	132	0.21
Positive	34	14			20	28	
**Lymph node invasion**							
Negative	192	74	0.007		143	123	0.003
Positive	29	25			17	37	

NOTE: Pearson's χ^2^ test, * Fisher's exact test. Abbreviations: α-fetoprotein, AFP; Carbohydrate antigen 19-9, CA19-9; Alanine transaminase, ALT; tumor lymph node metastasis, TNM

**Table 2:** Univariate analysis and multivariate analysis of prognosis factors associated with survival

Characteristics	Univariate analysis					Multivariate analysis			
	Cumulative recurrence rate		OS			Cumulative recurrence rate		OS	
	HR (95%CI)	*p*	HR (95%CI)	*p*		HR (95%CI)	*p*	HR (95%CI)	*p*
Age, year (>58 vs ≤58)	0.830(0.627-1.098)	0.192	0.935(0.718-1.218)	0.618		NA	NA	NA	NA
Gender (male vs female)	1.073(0.808-1.425)	0.627	1.144(0.873-1.498)	0.33		NA	NA	NA	NA
HBV infection (positive vs negative)	1.033(0.754-1.416)	0.838	0.884(0.661-1.181)	0.404		NA	NA	NA	NA
Liver cirrhosis (positive vs negative)	1.230(0.901-1.679)	0.192	1.184(0.883-1.589)	0.259		NA	NA	NA	NA
Hepatolithiasis (positive vs negative)	1.295(0.925-1.813)	0.132	2.041(1.587-2.625)	<0.001		NA	NA	NA	NA
Tumor differentiation (III/IV vs I/II)	1.296(0.977-1.719)	0.072	1.194(0.912-1.563)	0.196		NA	NA	NA	NA
Tumor size, cm (>5 vs ≤5)	1.422(1.073-1.884)	0.014	1.509(1.153-1.975)	0.003		1.298(0.972-1.733)	0.076	1.418(1.079-1.865)	0.012
Tumor number (multiple vs single)	1.814(1.323-2.488)	<0.001	1.632(1.207-2.206)	0.001		1.693(1.229-2.333)	0.001	1.541(1.135-2.094)	0.006
Child-Pugh classification (B vs A)	0.620(0.255-1.509)	0.293	0.893(0.420-1.896)	0.768		NA	NA	NA	NA
TNM stage (III vs I/II)	1.892(1.382-2.592)	<0.001	2.027(1.506-2.726)	<0.001		NA	NA	NA	NA
Microvascular invasion or Lymph node invasion (positive vs negative)	1.871(1.401-2.500)	<0.001	1.989(1.513-2.614)	<0.001		1.730(1.275-2.347)	<0.001	1.777(1.337-2.363)	<0.001
PD1(high vs low)	1.374(1.039-1.817)	0.026	1.392(1.067-1.816)	0.015		0.922(0.623-1.365)	0.685	1.130(0.791-1.614)	0.503
PD-L1(high vs low)	1.657(1.239-2.215)	0.001	1.420(1.073-1.880)	0.014		1.737(1.169-2.601)	0.007	1.268(0.874-1.842)	0.211
PD1/PD-L1 (double high vs others)	1.625(1.214-2.173)	0.001	1.406(1.061-1.862)	0.018		1.607(1.200-2.152)	0.001	1.364(1.028-1.811)	0.031

NOTE: Cox proportional hazards regression model. Abbreviation: NA, not applicable

## Abbreviations

ICC: intrahepatic cholangiocarcinoma
HCC: hepatocellular carcinoma
HBV: hepatitis B virus
PD1: programmed cell death protein 1
PD-L1: programmed cell death protein ligand 1
TMA: tissue microarray
IHC: immunohistochemistry
OS: overall survival

## References

[1] Torre LA, Bray F, Siegel RL, Ferlay J, Lortet-Tieulent J, Jemal A (2015). Global cancer statistics, 2012. CA Cancer J Clin.

[2] Zhou J, Sun HC, Wang Z, Cong WM, Wang JH, Zeng MS (2018). Guidelines for Diagnosis and Treatment of Primary Liver Cancer in China (2017 Edition). Liver Cancer.

[3] Liu ZY, Zhou YM, Shi LH, Yin ZF (2011). Risk factors of intrahepatic cholangiocarcinoma in patients with hepatolithiasis: a case-control study. Hepatobiliary Pancreat Dis Int.

[4] Wu J, Yang S, Xu K, Ding C, Zhou Y, Fu X (2018). Patterns and Trends of Liver Cancer Incidence Rates in Eastern and Southeastern Asian Countries (1983-2007) and Predictions to 2030. Gastroenterology.

[5] Mavros MN, Economopoulos KP, Alexiou VG, Pawlik TM (2014). Treatment and Prognosis for Patients With Intrahepatic Cholangiocarcinoma: Systematic Review and Meta-analysis. JAMA Surg.

[6] Sirica AE, Gores GJ, Groopman JD, Selaru FM, Strazzabosco M, Wang XW (2018). Intrahepatic Cholangiocarcinoma: Continuing Challenges and Translational Advances.

[7] de Jong MC, Nathan H, Sotiropoulos GC, Paul A, Alexandrescu S, Marques H (2011). Intrahepatic cholangiocarcinoma: an international multi-institutional analysis of prognostic factors and lymph node assessment. J Clin Oncol.

[8] Finn OJ (2008). Cancer immunology. N Engl J Med.

[9] Migden MR, Rischin D, Schmults CD, Guminski A, Hauschild A, Lewis KD (2018). PD-1 Blockade with Cemiplimab in Advanced Cutaneous Squamous-Cell Carcinoma. N Engl J Med.

[10] Le DT, Uram JN, Wang H, Bartlett BR, Kemberling H, Eyring AD (2015). PD-1 Blockade in Tumors with Mismatch-Repair Deficiency. N Engl J Med.

[11] Kansy BA, Concha-Benavente F, Srivastava RM, Jie HB, Shayan G, Lei Y (2017). PD-1 Status in CD8(+) T Cells Associates with Survival and Anti-PD-1 Therapeutic Outcomes in Head and Neck Cancer. Cancer Res.

[12] Chowell D, Morris LGT, Grigg CM, Weber JK, Samstein RM, Makarov V (2018). Patient HLA class I genotype influences cancer response to checkpoint blockade immunotherapy. Science.

[13] Wei SC, Levine JH, Cogdill AP, Zhao Y, Anang NAS, Andrews MC (2017). Distinct Cellular Mechanisms Underlie Anti-CTLA-4 and Anti-PD-1 Checkpoint Blockade. Cell.

[14] Topalian SL, Taube JM, Anders RA, Pardoll DM (2016). Mechanism-driven biomarkers to guide immune checkpoint blockade in cancer therapy. Nat Rev Cancer.

[15] Zou W, Chen L (2008). Inhibitory B7-family molecules in the tumour microenvironment. Nat Rev Immunol.

[16] Chen L (2004). Co-inhibitory molecules of the B7-CD28 family in the control of T-cell immunity. Nat Rev Immunol.

[17] Gao Q, Wang XY, Qiu SJ, Yamato I, Sho M, Nakajima Y (2009). Overexpression of PD-L1 significantly associates with tumor aggressiveness and postoperative recurrence in human hepatocellular carcinoma. Clin Cancer Res.

[18] Solinas A, Calvisi DF (2016). Programmed death ligand 1 expression in hepatocellular carcinoma: A prognostic marker and therapeutic target for liver cancer?. Hepatology.

[19] Havel JJ, Chowell D, Chan TA (2019). The evolving landscape of biomarkers for checkpoint inhibitor immunotherapy. Nat Rev Cancer.

[20] Ribas A, Hu-Lieskovan S (2016). What does PD-L1 positive or negative mean?. J Exp Med.

[21] Sato Y, Kinoshita M, Takemura S, Tanaka S, Hamano G, Nakamori S (2017). The PD-1/PD-L1 axis may be aberrantly activated in occupational cholangiocarcinoma. Pathol Int.

[22] Ye Y, Zhou L, Xie X, Jiang G, Xie H, Zheng S (2009). Interaction of B7-H1 on intrahepatic cholangiocarcinoma cells with PD-1 on tumor-infiltrating T cells as a mechanism of immune evasion. J Surg Oncol.

[23] Fontugne J, Augustin J, Pujals A, Compagnon P, Rousseau B, Luciani A (2017). PD-L1 expression in perihilar and intrahepatic cholangiocarcinoma. Oncotarget.

[24] Gani F, Nagarajan N, Kim Y, Zhu Q, Luan L, Bhaijjee F (2016). Program Death 1 Immune Checkpoint and Tumor Microenvironment: Implications for Patients With Intrahepatic Cholangiocarcinoma. Ann Surg Oncol.

[25] Khan SA, Thomas HC, Davidson BR, Taylor-Robinson SD (2005). Cholangiocarcinoma. Lancet.

[26] Bridgewater J, Galle PR, Khan SA, Llovet JM, Park JW, Patel T (2014). Guidelines for the diagnosis and management of intrahepatic cholangiocarcinoma. J Hepatol.

[27] Ishak KG, Anthony PP, Sobin LH (2012). Histological typing of tumours of the liver.

[28] Zhu AX, Finn RS, Edeline J, Cattan S, Ogasawara S, Palmer D (2018). Pembrolizumab in patients with advanced hepatocellular carcinoma previously treated with sorafenib (KEYNOTE-224): a non-randomised, open-label phase 2 trial. Lancet Oncol.

[29] Yang LX, Gao Q, Shi JY, Wang ZC, Zhang Y, Gao PT (2015). Mitogen-activated protein kinase kinase kinase 4 deficiency in intrahepatic cholangiocarcinoma leads to invasive growth and epithelial-mesenchymal transition. Hepatology.

[30] Shi GM, Ke AW, Zhou J, Wang XY, Xu Y, Ding ZB (2010). CD151 modulates expression of matrix metalloproteinase 9 and promotes neoangiogenesis and progression of hepatocellular carcinoma. Hepatology.

[31] Rimm DL, Han G, Taube JM, Yi ES, Bridge JA, Flieder DB (2017). A Prospective, Multi-institutional, Pathologist-Based Assessment of 4 Immunohistochemistry Assays for PD-L1 Expression in Non-Small Cell Lung Cancer. JAMA Oncol.

[32] Tyson GL, El-Serag HB (2011). Risk factors for cholangiocarcinoma. Hepatology.

[33] Su CH, Shyr YM, Lui WY, P'Eng FK (1997). Hepatolithiasis associated with cholangiocarcinoma. Br J Surg.

[34] Calvisi DF (2013). Inhibition of hepatitis B virus-associated liver cancer by antiplatelet therapy: a revolution in hepatocellular carcinoma prevention?. Hepatology.

[35] Tang LSY, Covert E, Wilson E, Kottilil S (2018). Chronic Hepatitis B Infection: A Review. JAMA.

[36] Harada K, Zen Y, Kanemori Y, Chen TC, Chen MF, Yeh TS (2001). Human REG I gene is up-regulated in intrahepatic cholangiocarcinoma and its precursor lesions. Hepatology.

[37] Labib PL, Goodchild G, Pereira SP (2019). Molecular Pathogenesis of Cholangiocarcinoma. BMC Cancer.

[38] Bally AP, Austin JW, Boss JM (2016). Genetic and Epigenetic Regulation of PD-1 Expression. J Immunol.

[39] Lei Z, Xia Y, Si A, Wang K, Li J, Yan Z (2018). Antiviral therapy improves survival in patients with HBV infection and intrahepatic cholangiocarcinoma undergoing liver resection. J Hepatol.

[40] Barnes E (2018). Unravelling the fate of functional PD1+ T cells in chronic viral hepatitis. J Clin Invest.

[41] Topalian SL, Hodi FS, Brahmer JR, Gettinger SN, Smith DC, McDermott DF (2012). Safety, activity, and immune correlates of anti-PD-1 antibody in cancer. N Engl J Med.

[42] Gehring AJ, Ho ZZ, Tan AT, Aung MO, Lee KH, Tan KC (2009). Profile of tumor antigen-specific CD8 T cells in patients with hepatitis B virus-related hepatocellular carcinoma. Gastroenterology.

[43] Samstein RM, Lee CH, Shoushtari AN, Hellmann MD, Shen R, Janjigian YY (2019). Tumor mutational load predicts survival after immunotherapy across multiple cancer types. Nat Genet.

[44] Pardoll DM (2012). The blockade of immune checkpoints in cancer immunotherapy. Nat Rev Cancer.

[45] Chew V, Lai L, Pan L, Lim CJ, Li J, Ong R (2017). Delineation of an immunosuppressive gradient in hepatocellular carcinoma using high-dimensional proteomic and transcriptomic analyses. Proc Natl Acad Sci U S A.

[46] Fung J (1961). Liver fluke infestation and cholangio-hepatitis. Br J Surg.

[47] Dey B, Kaushal G, Jacob SE, Barwad A, Pottakkat B (2016). Pathogenesis and Management of Hepatolithiasis: A Report of Two Cases. J Clin Diagn Res.

[48] Chisari FV, Ferrari C (1995). Hepatitis B virus immunopathogenesis. Annu Rev Immunol.

