# Supplementary material for: Distinct PD-L1/PD1 Profiles and Clinical Implications in Intrahepatic Cholangiocarcinoma Patients with Different Risk Factors
Source: Theranostics. 2019 Jul 9;9(16):4678–87. doi: 10.7150/thno.36276 (PMC6643449; doi:10.7150/thno.36276)
Supplement: Supplementary file 1 — Supplementary figures and tables. [file thnov09p4678s1.pdf]

Supplement Table 1 Clinicopathological characteristics of 320 ICC patients

Characteristics	Number [%]
**Age, y**	
≤58	169[52.8%]
>58	151[47.2%]
**Gender**	
Male	191[59.7%]
Female	129[40.3%]
**Risk factors-related ICC**	
HBV infection*	233[72.8%]
Hepatolithiasis	18[5.6%]
Undetermined[#]	76[23.8%]
**Liver cirrhosis**	
Negative	235[73.4%]
Positive	85[26.6%]
**HBV DNA copy number**	
Negative	133[41.6%]
Positive	27[8.4%]
Undetermined	160[50.0%]
**AFP,** ng/mL	
<20	280[87.5%]
≥20	40[12.5%]
**CA19-9**, U/mL	
<37	165[51.6%]
≥37	155[48.4%]
**ALT,** U/L	
<75	297[92.8%]
≥75	23[7.2%]
**Child-Pugh classification**	
A	309[96.6%]
B	11[3.4%]
**Tumor multiplicity**	
Single	245[76.6%]
Multiple	75[23.4%]
**Tumor encapsulation**	
Complete	37[11.6%]
None	283[88.4%]
**Tumor size,** cm	
≤5	144[45%]
>5	176[55%]
**Microvascular invasion**	
Negative	272[85.0%]
Positive	48[15.0%]

**Lymph node invasion**	
Negative	266[83.1%]
Positive	54[16.9%]
**Tumor differentiation**	
I/II	195[60.9%]
III/IV	125[39.1%]
**TNM stage**	
I/II	248[77.5%]
III	72[22.5%]

*HBV infection (hepatitis B virus infection) refers to HBcAb positive or HBsAg positive;

#Patients without HBV infection or hepatolithiasis;

Abbreviation: α-fetoprotein, AFP; carbohydrate antigen 19-9, CA19-9; alanine transaminase, ALT; tumor lymph node metastasis, TNM

TNM stage was assigned according to the “American Joint Committee on Cancer 8th edition”.

Supplementary Table 2 Clinicopathological characteristics of 7 advanced ICC patients with anti-PD1 immunotherapy

No.	Gender	Age	HBV infection	Times of anti-PD1 immunotherapy	PD1 expression	PD-L1 expression
1	Male	50	+	8	low	low
2	Male	49	+	11	low	low
3	Male	52	+	5	high	low
4	Female	55	+	6	high	low
5	Male	50	+	12	high	low
6	Male	53	+	4	high	high
7	Female	45	+	4	high	high

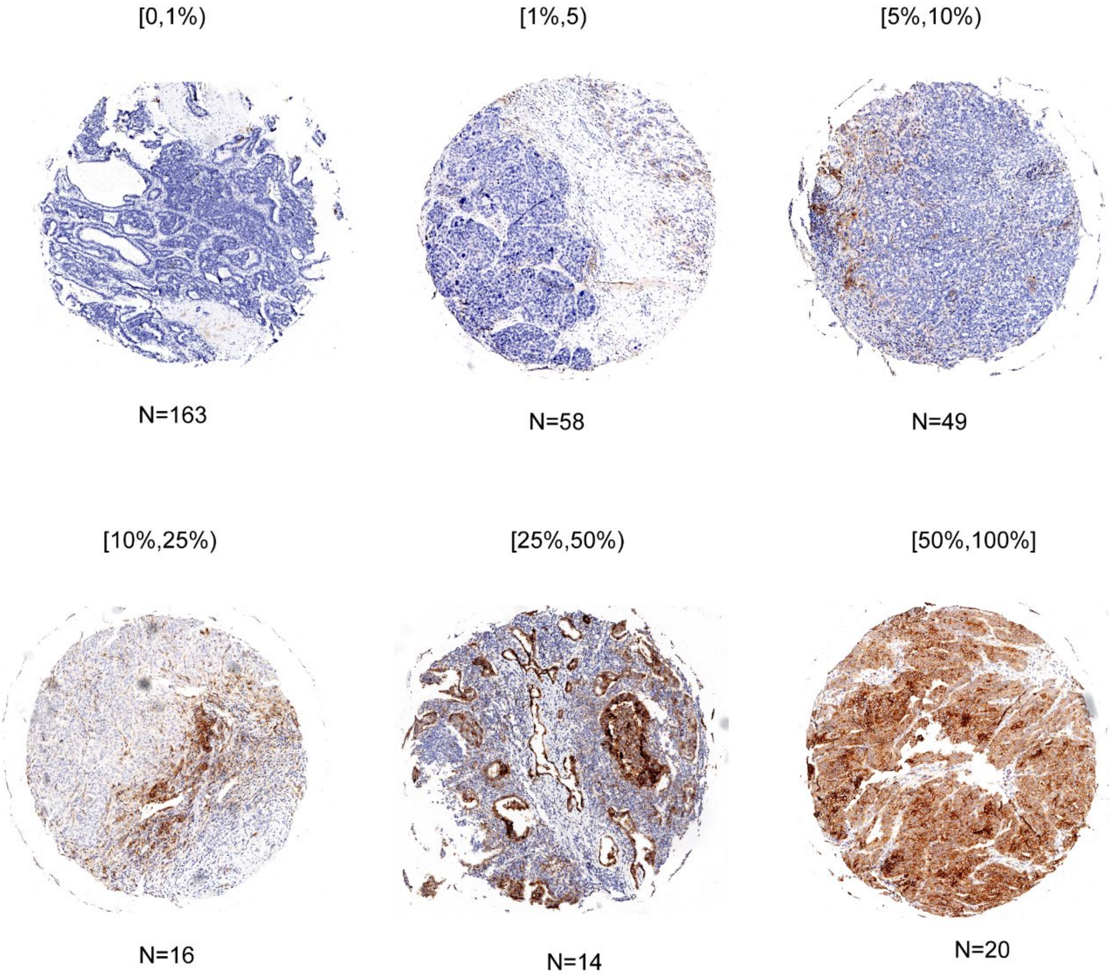

Supplementary Figure 1. PD-L1 expression on a 6-point scale.

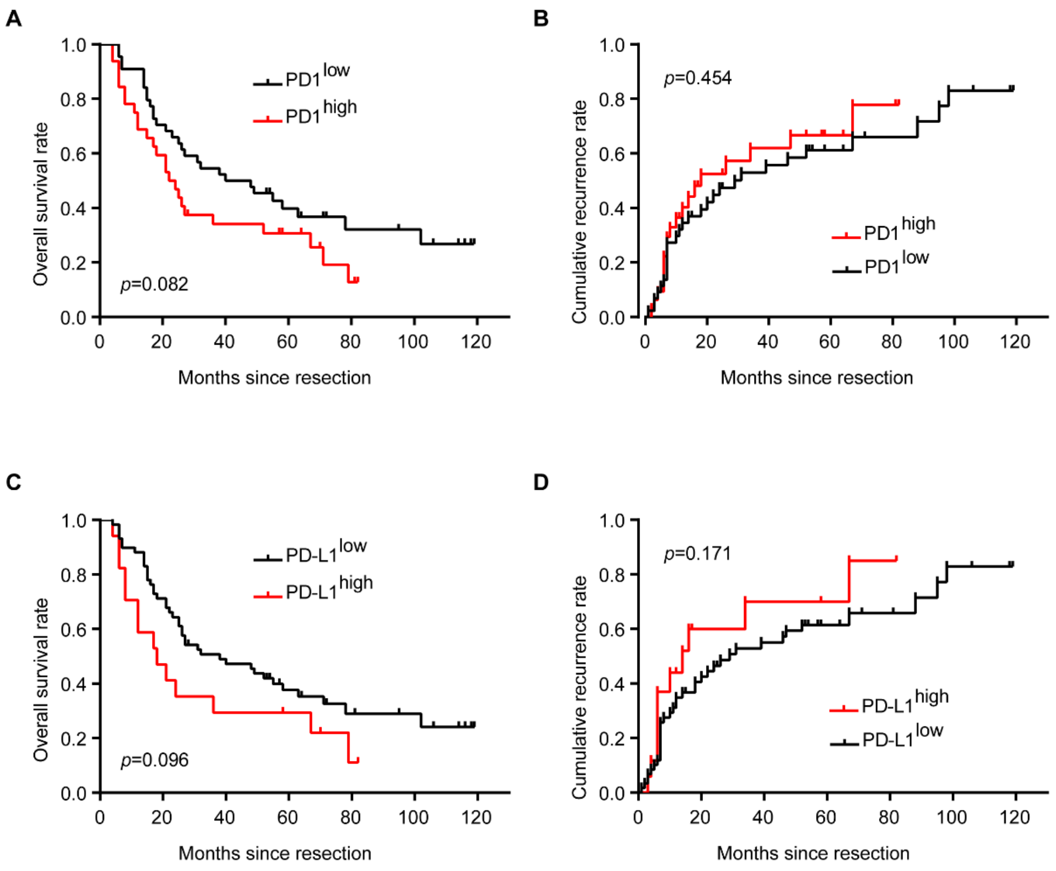

Supplementary Figure 2. Profiles and implication of PD1/PD-L1 expression in ICC patients with undetermined risk factors.

(**A**) Kaplan-Meier estimate of overall survival of patients with undetermined risk factors, grouped by PD1 level (log-rank test).

(**B**) Kaplan-Meier estimate of cumulative recurrence in patients with undetermined risk factors, grouped by PD1 level (log-rank test).

(**C**) Kaplan-Meier estimate of overall survival of patients with undetermined risk factors, grouped by PD-L1 level (log-rank test).

(**D**) Kaplan-Meier estimate of cumulative recurrence in patients with undetermined risk factors, grouped by PD-L1 level (log-rank test).
